# Novel method for bronchial stump coverage for prevents postpneumonectomy bronchopleural fistula: pedicled thymopericardial fat flap

**DOI:** 10.1186/s13019-022-02032-0

**Published:** 2022-11-11

**Authors:** Kenan Can Ceylan, Güntuğ Batıhan, Şeyda Örs Kaya

**Affiliations:** 1 The University of Health Sciences Dr Suat Seren Chest Diseases and Chest Surgery Training and Research Hospital, Izmir, Turkey; 2 Department of Thoracic Surgey, Kars State Hospital, Yenişehir, Ismail Aytemiz 55, 36002 Kars, Turkey

**Keywords:** Bronchopleural fistula, Pneumonectomy, Thymopericardial fat flap

## Abstract

**Background:**

Bronchopleural fistula (BPF) is a serious complication with high mortality and morbidity that can be seen after lung resections. Although several methods have been described to prevent postoperative BPF it is still unclear which method is the best. In this study, we have used tymopericardial fat flap (TPFF) to cover the bronchial stump in patients after pneumonectomy and aim to show its feasibility and efficacy to prevent BPF.

**Methods:**

Between January 2013 and June 2021, 187 patients with lung cancer underwent pneumonectomy at our institution. Among them, 53 patients underwent bronchial stump coverage with TPFF. In other 134 patients there wasn’t used any coverage method. Patient characteristics, preoperative status, surgical procedures, perioperative course, pathological findings, and long-term prognoses were evaluated retrospectively.

**Results:**

Postoperative BPF was observed in 16 (%8.5) patients. It was observed that TPFF was applied in only 1 of the patients who developed BPF. A statistically significant difference was detected between TPFF-coverage with non-coverage groups in terms of postoperative BPF rates (*p* = 0.044). Other factors associated with the development of postoperative BPF in univariate analysis were right sided pneumonectomy, and re-operation.

**Conclusion:**

Bronchial stump coverage with TPFF is a feasible and effective method to prevent postpneumonectomy BPF.

## Background

Bronchopleural fistula (BPF) is one of the most serious complications seen after lung resections and has been especially associated with pneumonectomy. The incidence of postpneumonectomy BPF varies between 4.5 and 20% in different series and it was shown that the incidence was higher in patients with right pneumonectomy, chronic obstructive pulmonary disease (COPD), a residual tumor on the bronchial stump, diabetes, and malnutrition. Besides patient-related risk factors, the surgical technique used for closure of the bronchial stump also has an impact on the occurrence of BPF [1–4].

The use of bronchial stump covering methods has been proposed to reduce the risk of developing bronchopleural fistulas, especially in the presence of risk factors. For this purpose, the usage of several autologous tissues like; omentum, pericardium, azygos vein, pericardial fat pad, thymus, diaphragm, intercostal muscle, and pleura were described in the literature however, there is no consensus on which technique is more successful in preventing BPF development [2, 5–7].

This retrospective study aims to evaluate the efficacy of the bronchial stump coverage with pedicled thymopericardial fat flap (TPFF) in patients who underwent pneumonectomy.

## Methods

### Patients

This retrospective study was approved by the local ethics committee (No. 49109414-604.02). Between January 2013 and June 2021, 1160 patients underwent anatomic lung resection for non-small cell lung cancer at our institution and 187 (%16.1) of these patients underwent pneumonectomy. Patients who underwent carinal and/or vascular sleeve pneumonectomy were excluded. Few patients who underwent bronchial stump coverage with the pericardial fat flap until the surgical technique fully became the “TPFF coverage” were not included in the study. Finally, 187 patients who met the inclusion criteria were included in the study.

For preoperative pulmonary risk assessment, spirometry, diffusing capacity for carbon monoxide (DLCO) and, if necessary, VO2max and cardiopulmonary exercise tests were performed for each patient. Thorax computed tomography (CT), and bronchoscopy was performed for all patients before surgery. In addition to these, Positron emission tomography (PET-CT) +/- endobronchial ultrasound-guided transbronchial needle aspiration (EBUS-TBNA) was performed in patients diagnosed with lung cancer. The preoperative mediastinal staging was performed in accordance with the National Comprehensive Cancer Network (NCCN) guidelines.

We investigated all 187 patients retrospectively and they were divided into two groups: those who underwent pneumonectomy plus bronchial stump coverage with thymopericardial fat flap (TPFF coverage group, n = 53) and those who underwent pneumonectomy without bronchial stump coverage (None-coverage group, n = 134).

While initially there was a tendency to apply TPFF to patients with a high risk of BPF, such as the presence of low serum albumin levels, comorbidities, history of neoadjuvant treatment, and right pneumonectomy, it has become a routine procedure over time regardless of the risk factors. Therefore, we do not have clear criteria for patient selection.

Patient characteristics, preoperative status, surgical procedures, perioperative course, pathological findings, and long-term prognoses were evaluated by review of the hospital records (Table 1).

**Table 1:** Perioperative and postoperative results of the patients

Variables	TPFF coverage (n = 53)	Non-coverage (n = 134)	*p* value
Age, years (mean ± SD)	62.83	60.60	0.064
Gender			0.108
Male	44 (83.0)	126 (94.0)	
Female	9 (17.0)	8 (6.0)	
Operative side [n (%)]			0.042
Left	45 (84.9)	94 (70.1)	
Right	8 (15.1)	40 (29.9)	
Co-morbidities [n (%)]			0.346
Diabetes	11 (20.8)	15 (11.2)	
Cardiac disorder	2 (3.8)	10 (7.5)	
Hypertension	4 (7.5)	6 (4.5)	
Multiple	3 (5.7)	7 (5.2)	
None	33 (62.3)	96 (73.1)	
FEV1 (lt)	2.14 ± 0.56	2.26 ± 0.56	0.187
FEV1 (%)	75.23 ± 14.25	84.43 ± 78.56	0.403
Preoperative serum albümine (mean ± SD)	4.28 ± 0.70	4.08 ± 0.45	0.023
Neoadjuvant therapy [n (%)]			0.176
No	35 (68.6)	105 (78.9)	
Yes	16 (31.4)	28 (21.1)	
Operation time (minutes) (mean ± SD)	224.53 ± 73.53	200.65 ± 95.91	0.101
Drainage time (days) (mean ± SD)	3.72 ± 2.24	3.92 ± 2.48	0.673
Hospitalization time (days) (mean ± SD)	5.92 ± 3.39	6.27 ± 3.23	0.592
Tumor size (mean ± SD)	5.21 ± 2.16	5.23 ± 2.31	0.951
Surgical margin to the main bronchus	2.51 ± 1.82	2.13 ± 1.42	0.180
Adjuvant chemotherapy [n (%)]			0.621
No	22 (41.5)	50 (37.6)	
Yes	31 (58.5)	83 (62.4)	
Adjuvant radiotherapy [n (%)]			0.424
No	33 (78.6)	65 (84.4)	
Yes	9 (21.4)	12 (15.6)	
Pathological stage [n (%)]			0.238
Stage I	8 (15.1)	11 (12.6)	
Stage II	15 (28.3)	37 (42.5)	
Stage III	30 (56.6)	39 (44.8)	
BPF [n (%)]			0.044
No	52 (98.1)	119 (88.8)	
Yes	1 (1.9)	15 (11.2)	

### Surgical technique

One-lung ventilation was performed in all patients with double-lumen intubation. An epidural catheter was routinely placed. An arterial line was placed in all cases for blood gas analyses and blood pressure monitoring. The subclavian or jugular catheter was placed at the side of the operation.

Patients were placed in the lateral decubitus position. Muscle sparing lateral thoracotomy or tri-portal video-assisted thoracoscopic surgery was performed. The VATS procedure was generally preferred in patients with small tumors with fissure invasion. Automatic stapling devices were used in 138 cases for the closure of the bronchus. Manual suturing was performed in only six patients and 3-0 polypropylene suture material was used.

Resection margins of the main bronchus were confirmed in patients with lung cancer by frozen section and an underwater test with sustained airway pressure of 30–35 mmH_2_O was performed in all patients to control air leakage.

After completion of the mediastinal lymph node dissection and air leakage control pericardial fat and ipsilateral thymus, the lobe was dissected with sharp and blunt dissection. First, the inferior part of the pericardial fat was dissected gently and freed from the diaphragm. Branches of the musculophrenic vessels could be dissected with energy devices. The dissection was continued anteriorly while retracting the fat pad posteriorly. Care should be taken not to damage the internal thoracic (IT) artery. To mobilize the pericardial fat pad, the middle and inferior pericardial branches of the IT artery must be dissected. When enough care is taken, the pericardial fat tissue can be easily removed over the pericardium.

**Fig. 1:**
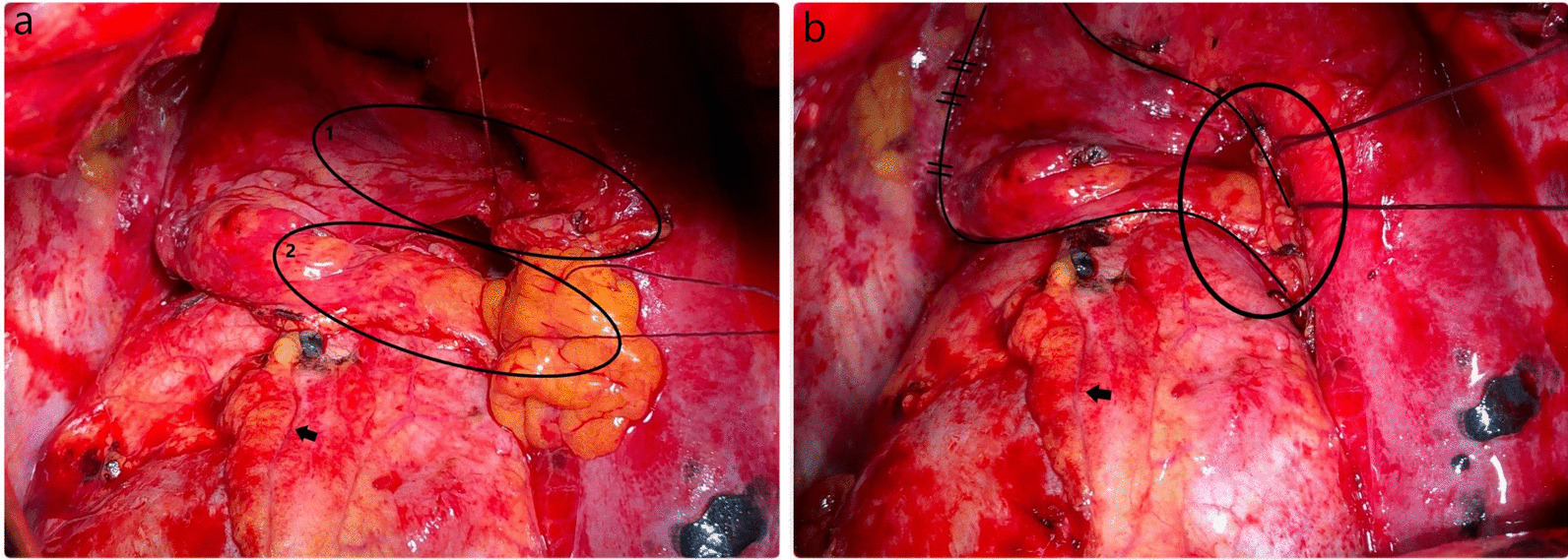
**a** Ipsilateral thymus lobe (1) was separated from the mediastinal pleura, the back surface of the sternum, and pericardium while preserving its vascular pedicle. The inferior part of the pericardial fat (2) was dissected gentle and freed from the diaphragm and pericardium. **b** Thymopericardial fat flap was sutured to the bronchial stump with interrupted horizontal mattress sutures

**Fig. 2:**
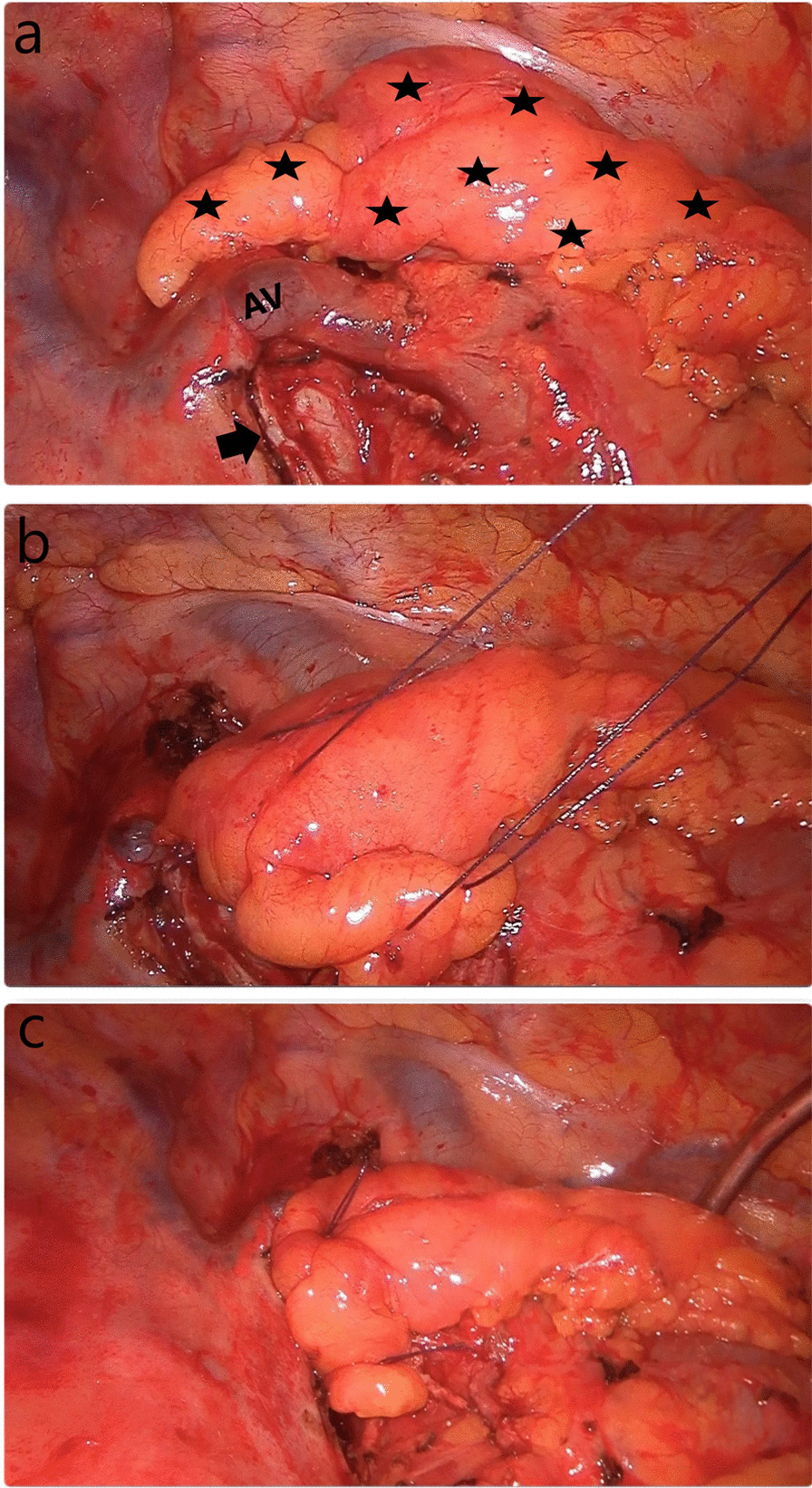
**a** Covering the right main bronchus with TPFF after right VATS pneumonectomy. Asterisks indicate dissected and prepared TPFF. The arrow indicates the stump of the right main bronchus. *AV* Azygos vein. **b** U-sutures passed through the peribronchial tissue and flap are seen. In order not to create extra tension on the stump, it would be more appropriate to pass the sutures through the peribronchial tissues instead of the bronchial cartilage. **c** Image of the thoracic cavity after completion of TPFF coverage

The ipsilateral thymus lobe was separated from the mediastinal pleura, the back surface of the sternum, and pericardium while preserving its vascular pedicle. Hereby, the pericardiophrenic artery, thymic and superior (sometimes middle) pericardial branches were preserved, and approximately 20 cm long, 5 cm wide, well-vascularized thymopericardial fat flap was obtained. While the TPFF is being created, the dissection can be performed using conventional surgical instruments, or it can be done with energy devices that combine ligation and division features. In our cases, we performed most of the dissections using an energy device to minimize the risk of postoperative hemorrhage. The TPFF was sutured to the bronchial stump with interrupted horizontal mattress sutures of 2-0 Vicryl (Figs. 1 and 2). Excessive dissection should be avoided in order not to disrupt the vascularity of the flap, as well should not be too much tension on the pedicle and the bronchial stump. TPFF coverage technique can be applied also via VATS with the same steps.

### Postoperative follow-up

We have preferred to keep the patients in the intensive care unit (ICU) for the first 24 h after the operation. The nutritional status of the patients was closely monitored, and nutritional support was started for each patient in the postoperative period. After the chest tube removal, patients who were in stable condition and enabled self-maintenance of normal daily activities were discharged. All patients had regular follow-up visits for 3 months for the first 2 years, then every 6 months up to 5 years.

A postoperative complication was defined as any deviation from the normal postoperative course. The bronchopleural fistula was confirmed bronchoscopically in all cases. Operative mortality was defined as death occurring within 30 days post-surgery.

### Statistical analysis

SPSS 25.0 (SPSS Inc., Chicago, IL, USA) was used to perform statistical analysis. Chi-squared or Fischer’s exact tests were used to compare frequencies in categorical variables. Normality of distribution was tested with the Shapiro-Wilk test for all numerical variables. Means and standard deviations were used in variables with the normal distribution. Non-normal distributed values were expressed as medians and interquartile ranges.

Continuous variables, expressed as mean value ± standard deviation (SD), were compared by unpaired Student’s t-tests. Because there are few events (BPF) compared with the number of predictors we preferred to perform logistic regression analyses with Firth’s correction. DFS and OS were estimated using the Kaplan-Meier method. Statistical significance was set at *p*-value < 0.05 (All *p* values presented were 2-sided).

## Results

From January 2013 to June 2021 a total of 187 patients fitted the criteria for inclusion in this retrospective study. There were 170 men and 17 women. The median age was 62 years, with a range of 31–80. Among 187 patients, 48 (%25.7) patients underwent right, 139 (%74.3) patients underwent left pneumonectomy.

The coverage of the bronchial stump with TPFF was performed in 53 (%28.3) patients. In 134 (%71.7) patients, there wasn’t used bronchial coverage method.

The distribution of characteristic features between TPFF coverage and the non-coverage group are summarized in Table 1. The operative side and mean preoperative serum albumin level were the characteristics that were statistically different between the two groups.

After the operation, a routine follow-up was performed in all cases and the mean follow-up time was 30.4 ± 26.54 months.

A statistically significant difference was detected between TPFF-coverage with non-coverage groups in terms of postoperative BPF rates (*p* = 0.044).

BPF was detected in 8 (% 16.7) of 48 patients who underwent right pneumonectomy, while it was detected in 8 (% 5.8) of 139 patients who underwent left pneumonectomy (*p* = 0.033).

Interestingly, the frequency of the need for reoperation was found to be statistically significantly higher than in patients with non-BPF (as shown in Table 2). There was no significant difference between patients with and without BPF in terms of age, gender, comorbidity, preoperative serum albumin level, neoadjuvant or adjuvant therapy status, and stage.

**Table 2:** Univariate analyses of risk factors for BPF

Risk factor	BPF (%) (n = 16)	No BPF (%) (n = 171)	*p*-value
Age, years (mean ± SD)	61.38 ± 7.78	61.21 ± 7.43	0.935
Gender			0.338
Male	16 (100.0)	154 (90.1)	
Female	0 (0)	17 (9.9)	
Albumin (mean ± SD)	4.14 ± 0.41	4.14 ± 0.55	0.997
Comorbidity			0.398
Yes	3 (18.8)	55 (32.2)	
No	13 (81.3)	116 (67.8)	
Operation side			0.033
Right	8 (50.0)	40 (23.4)	
Left	8 (50.0)	131 (76.6)	
Neoadjuvant therapy [n (%)]			0.765
Yes	3 (18.8)	41 (24.4)	
No	13 (81.3)	127 (75.6)	
Adjuvant chemotherapy [n (%)]			0.428
Yes	9 (56.3)	105 (61.8)	
No	7 (43.8)	65 (38.2)	
Stage			0.201
I	5 (31.3)	29 (16.9)	
II	3 (18.8)	65 (37.8)	
III	8 (50.0)	78 (45.3)	
Surgical approach			0.151
VATS	0 (0.0)	20 (11.7)	
Thoracotomy	16 (100.0)	151 (88.3)	
Re-operation			0.043
Yes	3 (18.8)	7 (4.1)	
No	13 (81.3)	164 (95.9)	
TPFF			0.044
Yes	1 (6.3)	52 (30.4)	
No	15 (93.8)	119 (69.6)	

Logistic regression analyses with Firth’s correction were performed to demonstrate risk factors for postoperative BPF. However, none of the parameters included in the modeling reached statistical significance (Table 3).

**Table 3:** Firth’s bias-reduced logistic regression analyses of the risk factors for BPF

Covariates	Estimate	Standard error	*p*-value	X^2^	95% Cl
TPF (0: no, 1: yes)	− 1.011	0.857	0.256	1.596	− 3.257–0.489
Operation side (0: left, 1: right)	0.881	0.541	0.115	2.478	− 0.223–1.967
Re-operation (0: no, 1: yes)	1.000	0.768	0.202	1.631	− 0.583–2.419
Surgical approach (0: thoracotomy, 1: VATS)	− 1.072	1.445	0.402	0.702	− 5.955–1.104

According to the results of long-term follow-up, the mean overall survival was 62.7 ± 3.7 months, and the disease-free survival was 39.0 ± 2.9 months.

## Discussion

Despite advances in surgical technique and postoperative care, pneumonectomy is still associated with high mortality and morbidity. Therefore, patients who are candidates for pneumonectomy should be evaluated in detail for their suitability for bronchovascular sleeve resection and the lung parenchyma should be spared as much as possible. In our series, the rate of pneumonectomy in all anatomic lung resections was %16.1. This rate is between %4.5 and %33 in various publications [8–10].

Bronchopleural fistula is one of the most severe complications seen after lung resections and is reported to be in the range of 4.5–20% after pneumonectomy. It is associated with increased mortality and morbidity, prolonged hospitalization, and higher costs. Various risk factors have been described for the development of BPF like; right-sided pneumonectomy, excessive dissection of the peribronchial structures, long bronchial stump, preoperative radiotherapy, positive sputum culture, low serum albumin level, and male gender [1–3]. In our study, in accordance with the literature, the rate of BPF was found to be higher in patients who underwent right pneumonectomy compared to the left.

Interestingly, the presence of reoperation was found to be associated with an increased postoperative BPF rate in univariate analyzes in our study (*p* = 0.043). A total of 10 patients underwent reoperation due to postoperative hemorrhage, and 3 of these patients developed BPF. We do not have enough data to reveal the relationship between reoperation and the risk of postoperative BPF. However, excessive blood transfusion, hypovolemia, prolonged intubation, and physiological stress caused by reoperation can be considered possible risk factors.

The difficult and time-consuming nature of BPF treatment has increased the importance of methods to prevent this complication. For this purpose, various methods of bronchial reinforcement have been described in the literature. Omentum, azygos vein, pericardium, a pedicled pericardial fat pad, intercostal muscle, pleura, and diaphragm have been used to increase the vascularity of the bronchial stump. Despite the variety of tissues that can be used, the issue of which method is the best is controversial [6, 11].

The use of a pedicled intercostal muscle flap is reported to have the potential risk of the development of heterotopic ossification leading to necrosis of the flap. Coverage of the bronchial stump with the pedicled pericardial flap requires to make a pericardiotomy and reconstruction of the pericardium. The omental flap is one of the most effective techniques for the prevention of bronchial fistula, but it requires an additional incision into the abdomen and diaphragm [12, 13].

The pericardial fat pad is another useful tissue for bronchial stump coverage. It was also shown that various angiogenic and growth factors are produced from the pericardial fat pad and help the healing of the bronchial stump [10, 14, 15]. However, in some patients, the volume and vascularization of this tissue flap are insufficient. Thus, we preferred to use the TPFF as a well-vascularized, voluminous tissue flap to cover the bronchial stump. The usage of the pedicled thymus flap for protection of the bronchial stump or bypass graft was described before and considered as a safe and effective alternative to the other tissue flaps. Ohtsuka et al. [16] have applied a thymopericardial fat flap (also called a pedicled prepericardial fat flap) to reinforce the vascular graft in 245 cases who underwent coronary artery bypass graft surgery. In another study, Wurts et al. [17] used TPFF in 5 patients with tracheal reconstruction and they called this flap the “thoracic omentum”, referring to the efficacy and usefulness of the omental flap.

In this study, we applied TPFF in 53 patients with pneumonectomy, and no postoperative BPF was detected. In univariate analyses, the BPF rate was significantly lower in patients who underwent bronchial coverage with TPFF (*p* = 0.044).

However, in multivariate analysis, none of the variables included in the modeling reached statistical significance (Table 3).

For thoracic surgeons with thymectomy experience, dissecting the ipsilateral thymus lobe and including it in the pericardial fatty tissue flap is not a challenging procedure. Moreover, TPFF coverage could be easily performed also via the VATS technique. Video thoracoscopy facilitates the detection and control of minor hemorrhages by providing better exposure in the anterior mediastinum. We performed VATS pneumonectomy in 20 patients and TPFF coverage was applied in 12 of them.

Although the mean operative time was found to be slightly increased in patients who underwent TPFF coverage, this difference was not statistically significant (*p* = 0.101). After a certain adaptation period, the preparation of the thymopericardial flap and its suturing to the bronchial stump can be performed in as little as 15 min.

**Fig. 3:**
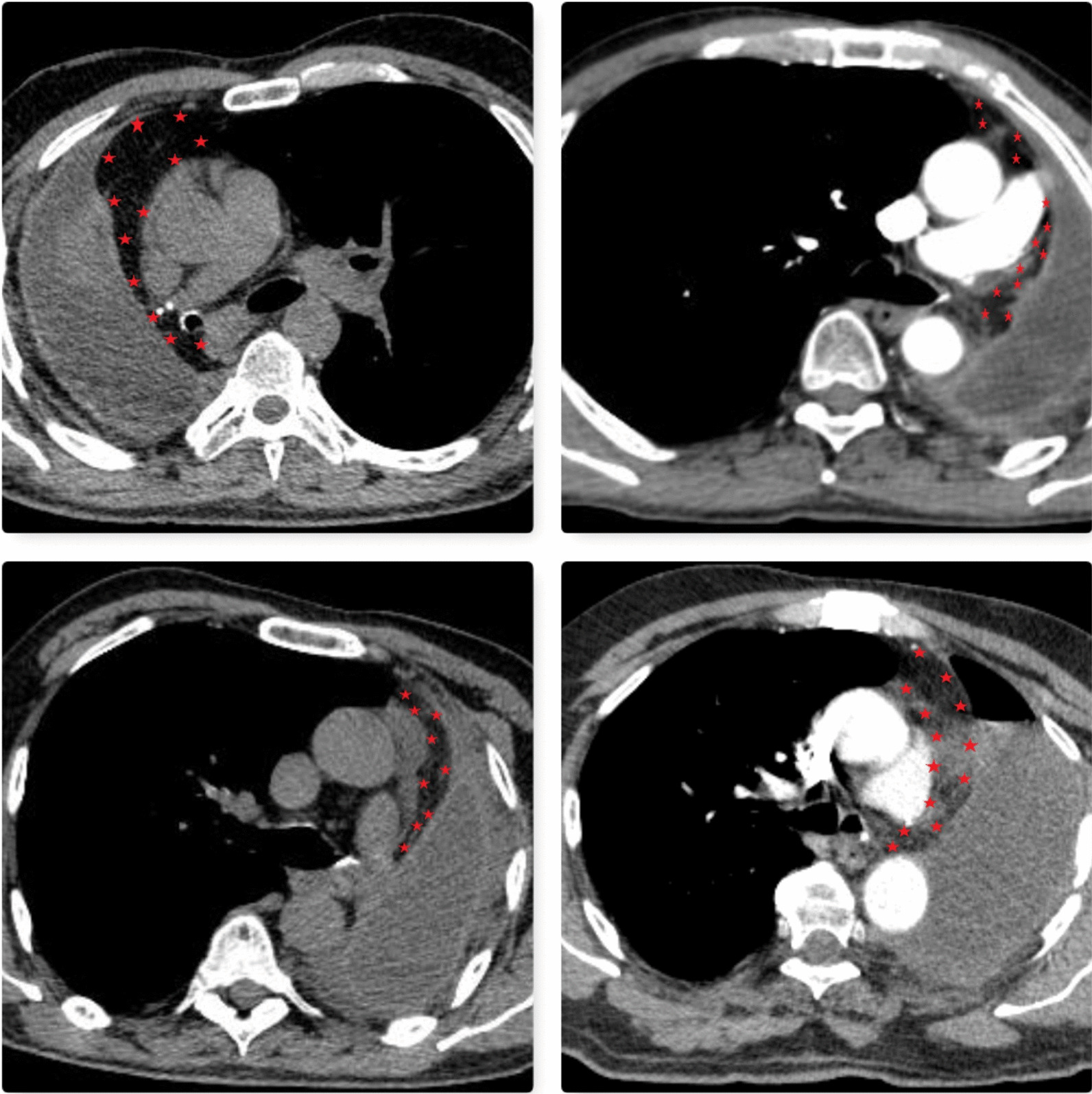
TPFF could be seen in thorax CT even after 6–12 months after operation (asterisks)

Interestingly, we detected the pedicled tissue flap around the bronchial stump could be in thorax CT even after 6–12 months in patients who underwent TPFF coverage (Fig. 3). Nagashima et al. [18] examined the postoperative change of free pericardial fat pad affixed to the bronchial stump and found a significant reduction in the volume of adipose tissue at the end of 6 months. Considering that bronchopleural fistula may occur in some patients in the very late period, we think that it would be more appropriate to use pedicle flaps instead of free harvested tissue.

This study had some limitations. Because of its retrospective nature, bias regarding patient selection existed. Although there was a tendency to apply TPFF in those with a high risk of BPF the criteria for usage of a TPFF were not defined clearly. One of the reasons for this is that while the TPFF application was initially decided on a patient-specific basis, it later became a routine procedure. Furthermore, to demonstrate the efficacy and superiority of the TPFF, it is necessary to compare it with other coverage methods, especially via large-scale prospective studies.

## Conclusion

Bronchial stump coverage with TPFF is an easy and feasible method to prevent postpneumonectomy BPFs and is also can be applied with VATS or thoracotomy. In this study BPF rate was significantly low in-patient group with TPFF. We think that it will be a good alternative to traditional bronchial stump coverage methods with its better vascularity and voluminous structure.

## Data Availability

The data underlying this article cannot be shared publicly due to the privacy of individuals that participated in the study. The data will be shared on reasonable request to the corresponding author.

## References

[1] Alpert JB, Godoy MC, Degroot PM, Truong MT, Ko JP (2014). Imaging the post-thoracotomy patient: anatomic changes and postoperative complications. Radiol Clin North Am.

[2] Hu XF, Duan L, Jiang GN, Wang H, Liu HC, Chen C (2013). A clinical risk model for the evaluation of bronchopleural fstula in nonsmall cell lung cancer after pneumonectomy. Ann Thorac Surg.

[3] Wright CD, Wain JC, Mathisen DJ, Grillo HC (1996). Postpneumonectomy bronchopleural fstula after sutured bronchial closure: incidence, risk factors, and management. J Thorac Cardiovasc Surg.

[4] DiMario M, Perrone F, Deschamps C, Rocco G (2015). A meta-analysis of the impact of bronchial stump coverage on the risk of bronchopleural fistula after pneumonectomy. Eur J Cardiothorac Surg.

[5] Klepetko W, Taghavi S, Pereslenyi A, Birsan T, Groetzner J, Kupilik N (1999). Impect of different coverage techniques on incidence of postpneumonectomy stump fistula. Eur J Cardiothorac Surg.

[6] Taghavi S, Marta GM, Lang G, Seebacher G, Winkler G, Schmid K (2005). Bronchial stump coverage with a pedicled pericardial flap: an effective method for prevention of postpneumonectomy bronchopleural fistula. Ann Thorac Surg.

[7] Linder M, Hapfelmeire A, Morresi-Hauf A, Schmid M, Hats R, Winter H (2010). Bronchopleural stump coverage and postpneumonectomy bronchopleural fistula. Asian Cardiovasc Thorac Surg.

[8] Sartipy U (2009). Prospective population-based study comparing quality of life after pneumonectomy and lobectomy. Eur J Cardiothorac Surg.

[9] Stéphan F, Boucheseiche S, Hollande J, Flahault A, Cheffi A, Bazelly B (2000). Pulmonary complications following lung resection: a comprehensive analysis of incidence and possible risk factors. Chest.

[10] Matsuoka K, Imanishi N, Yamada T, Matsuoka T, Nagai S, Ueda M (2016). Clinical results of bronchial stump coverage using free pericardial fat pad. Interact Cardiovasc Thorac Surg.

[11] Llewellyn-Bennett R, Wotton R, West D (2013). Prophylactic flap coverage and the incidence of bronchopleural fistula after pneumonectomy. Interact CardioVasc Thorac Surg.

[12] Anderson TM, Miller JI (1995). Use of pleura, azygos vein, pericardium, and muscle fl aps in tracheobronchial surgery. Ann Thorac Surg.

[13] D’Andrilli A, Ibrahim M, Andreetti C, Ciccone AM, Venuta F, Rendina EA (2009). Transdiaphragmatic harvesting of the omentum through thoracotomy for bronchial stump reinforcement. Ann Thorac Surg.

[14] Shoji F, Yano T, Miura N, Morodomi Y, Yoshida T, Onimaru M (2011). Pericardial fat pad tissue produces angiogenic factors for healing the bronchial stump. Interact CardioVasc Surg.

[15] Ichinose Y, Asoh H, Yano T, Yokoyama H, Inoue T, Tayama K (1995). Use of a pericardial fat pad flap for preventing bronchopleural fistula: an experimental study focusing on the angiogenesis and cytokine production of the fat pad. Surg Today.

[16] Ohtsuka T, Ninomiya M, Nonaka T (2009). Thymopericardial augmented encasement for coronary artery bypass graft surgery: a report of 245 cases. Innovations (Phila).

[17] Wurtz A, Juthier F, Conti M, Vincentelli A (2011). The “thymopericardial fat flap”: a versatile flap in thoracic and cardiovascular surgery. J Thorac Cardiovasc Surg.

[18] Nagashima T, Ito H, Samejima J, Nemoto D, Eriguchi D, Nakayama H, Woo T, Masuda M (2019). Postoperative changes of the free pericardial fat pad for bronchial stump coverage. J Thorac Dis.

